# Risk-benefit analysis of tuberculosis infection testing for household contact management in high-burden countries: a mathematical modelling study

**DOI:** 10.1016/S2214-109X(20)30075-9

**Published:** 2020-04-27

**Authors:** Courtney M Yuen, James A Seddon, Salmaan Keshavjee, Peter J Dodd

**Affiliations:** a Division of Global Health Equity, Brigham and Women's Hospital, Boston, MA, USA; b Department of Global Health and Social Medicine, Harvard Medical School, Boston, MA, USA; c Department of Infectious Diseases, Imperial College London, London, UK; d Desmond Tutu TB Centre, Department of Paediatrics and Child Health, Stellenbosch University, Cape Town, South Africa; e School of Health and Related Research, University of Sheffield, Sheffield, UK

## Abstract

**Background:**

Preventive therapy for tuberculosis reduces the risk of disease in people who have been infected but who are not sick. Countries with a high burden of tuberculosis that are expanding preventive therapy use must decide how tuberculosis infection testing should be used for risk stratification among household contacts of patients with tuberculosis.

**Methods:**

We modelled the risks of tuberculosis disease and severe adverse events, comparing the following two preventive therapy strategies: preventive therapy for all household contacts, or preventive therapy for only household contacts with a positive tuberculin skin test (TST) result. We used data from clinical trials and literature on tuberculosis natural history to model outcomes, assuming different preventive therapy regimens, ages, and TST positivity prevalence.

**Findings:**

Assuming 25% prevalence of TST positivity among 1000 household contacts aged 0–17 years, a treat-all approach with isoniazid and rifapentine compared with a treat-TST-only approach led to 13 fewer incident tuberculosis cases (IQR −5 to −18) and four additional severe adverse events (2 to 6). With rifampicin, the difference was 11 fewer incident tuberculosis cases (–3 to −17) and two additional severe adverse events (1 to 3). For adults, a treat-all approach led to fewer incident tuberculosis cases, and additional adverse events increased with age. Assuming 25% prevalence of TST positivity among adult contacts, a treat-all approach would lead to around two fewer tuberculosis cases per 1000 contacts for all regimens; the number of additional severe adverse events ranged from seven (IQR 5 to 8) for 18 to 34-year-olds treated with rifampicin to 63 (50 to 74) for people older than 64 years treated with isoniazid and rifapentine. A rifampicin-only regimen was associated with the fewest additional severe adverse events (seven [IQR 5 to 8] per 1000 adults aged 18–34 years and 35–64 years, and 17 [9 to 23] per 1000 adults older than 64 years).

**Interpretation:**

Based on the available data, giving preventive therapy to all household contacts would probably reduce the incidence of tuberculosis cases in high-burden settings. Adverse events could be minimised by using non-isoniazid regimens and, in adults older than 18 years, focusing treatment on individuals with a positive infection test.

**Funding:**

Bill & Melinda Gates Foundation, UK Medical Research Council, and UK Department for International Development.

## Introduction

An estimated 10 million people develop tuberculosis globally every year, including 1 million children.^1^ When a person is infected with the bacterium that causes tuberculosis, disease might develop rapidly or after a period of time, with the highest risk of disease occurring in the first 1–2 years.^2^ If a person is infected but has not yet developed disease, the infection can be treated with preventive therapy. Clinical trials have established the effectiveness of preventive therapy to be 60–70% in reducing the risk of developing tuberculosis disease.^3^ Mathematical modelling suggests that along with active case-finding and effective treatment, preventing the development of tuberculosis disease is a critical component of a comprehensive global tuberculosis strategy, without which tuberculosis elimination cannot be achieved.^4^, ^5^

Close contacts of patients with tuberculosis, particularly those in the same household, are a priority group for preventive therapy given the high probability of recent infection. In low-income and middle-income countries, around 45% of household contacts have been found to have latent tuberculosis infection on average.^6^ Best practice for managing household contacts of a newly diagnosed tuberculosis patient involves prompt evaluation of all household members, treatment of those with tuberculosis disease, and provision of preventive therapy to those who do not have tuberculosis disease but who are at risk of developing disease in the future.^7^ Guidelines for risk stratification to decide who should get preventive therapy vary across countries.^8^ In settings with low tuberculosis incidence, preventive therapy is generally offered to contacts who test positive for tuberculosis infection, as well as others at particularly high risk of disease progression. In many settings with high tuberculosis incidence, guidelines recommend preventive therapy for only contacts younger than 5 years and people living with HIV—the perceived highest-risk groups—without testing for tuberculosis infection. However, it is estimated that globally, if all household contacts younger than 15 years were evaluated and given preventive therapy if infected but not sick, 160 000 tuberculosis cases among children could be averted annually.^9^ Reductions in morbidity would be even greater if adult household contacts with tuberculosis infection were also treated.^5^

**Research in context**

**Evidence before this study**

Systematic reviews have quantified the effectiveness of preventive therapy for reducing the risk of tuberculosis disease. Mathematical modelling studies have projected that elimination of tuberculosis globally will require increased use of preventive therapy. A systematic review quantified the risks of adverse events associated with different preventive therapy regimens, and several studies have reported that adverse event risk—particularly for hepatotoxicity—increases with age. To determine whether these data had been combined in a mathematical model to compare the risks and benefits of preventive therapy, we did a PubMed search on Oct 24, 2019, with no language restrictions, for studies published since inception, combining the tuberculosis infection terms (“latent tuberculosis” OR “tuberculosis infection” OR “TB infection” OR “preventive therapy”) with variants of the term “mathematical modelling”. This search yielded 98 articles, of which 25 reported modelling of preventive therapy interventions. Only one of these included adverse events as an outcome, comparing incident tuberculosis cases and hepatitis events that would be expected if people over 60 years old in Hong Kong were tested for latent tuberculosis infection and treated with isoniazid.

**Added value of this study**

To our knowledge, this study is the first to model both incident tuberculosis cases and adverse events associated with different preventive therapy strategies for contact management in high-burden settings. We separately modelled these outcomes for different age groups and different preventive therapy regimens, and we compared strategies of treating all contacts and treating only contacts with positive tuberculin skin tests. We incorporate in our model systematic review evidence on the efficacy of different preventive therapy regimens and age-stratified adverse event risk data from recent trials of rifapentine-based and rifampicin-based regimens.

**Implications of all the available evidence**

In settings with high tuberculosis burdens, expanding use of preventive therapy to all contacts will help to accelerate declines in tuberculosis incidence. For contacts younger than 18 years, tuberculosis infection testing might not be necessary, as treating all contacts in this age group with preventive therapy would lead to substantially more protection with minimal additional adverse events compared with giving preventive therapy to only those with confirmed infection. When expanding preventive therapy to older contacts, adverse events are more of a concern; however, these can be minimised by using a rifampicin-only regimen, focusing treatment on those with a positive infection test, and actively monitoring patients at higher risk of adverse events via regular evaluation of signs and symptoms and liver function testing.

Given the importance of tuberculosis infection treatment in combating the global tuberculosis epidemic, WHO released updated guidance in 2018 supporting expanded use of preventive therapy in countries with high tuberculosis burdens.^7^ As many of these countries consider revising national guidelines to expand use of preventive therapy beyond young children and people living with HIV, the question arises of whether tuberculosis infection testing should be used for risk stratification among contacts, as is done in low-burden settings. Available tests for tuberculosis infection include skin tests (most commonly the tuberculin skin test [TST]) and interferon gamma release assay (IGRA) blood tests. Both types of test detect an immune response to *Mycobacterium tuberculosis* antigens but cannot distinguish between current infection and an infection cleared by the immune system. Both can give false negative results in people with compromised immune systems or very recent infection, and the two tests can give discordant results.^10^ Given their imperfect sensitivity,^10^ the negative predictive value of the tests for detecting tuberculosis infection is restricted where prevalence of tuberculosis infection is high. Moreover, both types of test have only partial value in predicting future tuberculosis disease.^11^ Finally, logistical barriers have limited the use of these tests in resource-poor settings—the TST requires cold-chain storage and two visits to complete, the IGRA requires a blood sample and laboratory infrastructure, and both require trained health staff to implement.

Given both diagnostic limitations and logistical challenges associated with tuberculosis infection testing in high-burden settings, an alternative approach would be to give preventive therapy to all household contacts once disease has been ruled out, as is done for young children and people living with HIV. However, preventive therapy can cause adverse events; therefore, it is necessary to weigh the risk that a person will develop tuberculosis against the risk of adverse events and ensure that the risk–benefit assessment is favourable. Multiple factors affect this risk–benefit assessment. Both the risk of developing tuberculosis disease and the risk of adverse events associated with preventive therapy vary by age.^2^, ^12^ Moreover, risks of adverse events are different for the four currently recommended preventive therapy regimens for drug-susceptible tuberculosis infection.^7^

There is a lack of clear guidance available to enable high-burden settings to develop contact management policies that expand the use of preventive therapy beyond young children and people living with HIV. To help fill this knowledge gap, we did a modelling exercise comparing the risk of future tuberculosis disease progression and the risk of severe adverse events associated with the following two preventive therapy strategies: giving preventive therapy to all household contacts of patients with tuberculosis or giving preventive therapy to only TST-positive household contacts.

## Methods

### Model framework

**Figure 1:**
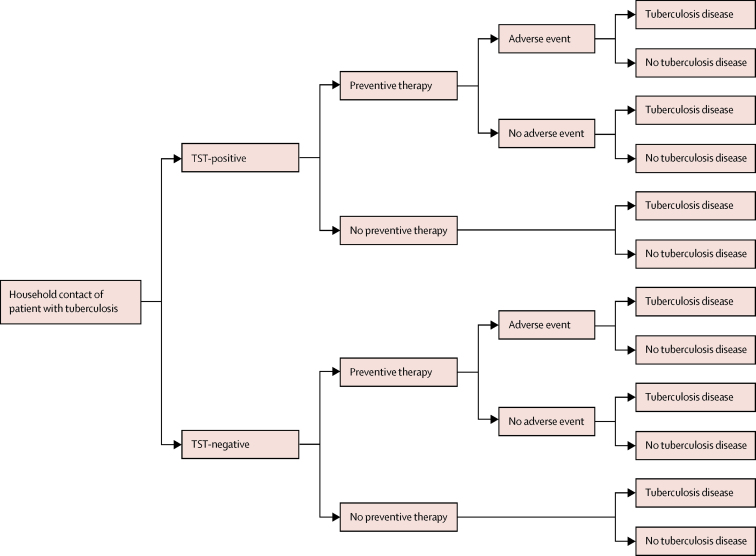
Model decision tree for tuberculosis disease and severe adverse event outcomes TST=tuberculin skin test.

To robustly explore uncertainty around individual-level benefits and harms and to avoid assumptions about transmission epidemiology, we used a decision tree model framework (figure 1). Risk of tuberculosis disease progression was dependent on TST status and receipt of preventive therapy, and anyone who received preventive therapy had a risk of severe adverse events. We did separate analyses for different age groups since the risk of disease progression and severe adverse events vary by age.^2^, ^12^ We did separate analyses for different preventive therapy regimens for which appropriate data were available, including 3 months of weekly isoniazid and rifapentine, 4 months of daily rifampicin, and 6 months of daily isoniazid. Appropriate data were unavailable for the 3 months of daily isoniazid and rifampicin regimen, the 1 month of daily isoniazid and rifapentine regimen, or regimens for drug-resistant tuberculosis. Modelling and analyses were done using R version 3.6. All code and data are available at the GitHub repository.

### Interventions and outcomes

For each age group and regimen, we compared two strategies: one in which all household contacts received preventive therapy (treat all), and one in which all household contacts were tested for tuberculosis infection by TST, but only those with a positive TST result received preventive therapy (treat TST-positive only). We assessed the following two outcomes of interest: incident tuberculosis disease and severe adverse events. Tuberculosis disease was considered a final endpoint, after which we did not consider whether treatment of tuberculosis disease might incur adverse events or cause additional disease or infection via transmission. Severe adverse events were conceptualised as the equivalent of grade 3 or higher adverse events based on standardised grading criteria used in clinical trials. Grade 3 adverse events are not life-threatening but are severe enough to be disabling or to warrant hospitalisation; grade 4 adverse events are life-threatening.^13^

### Model parameters

Table 1 summarises the parameters used in the model. We sought to obtain age-stratified estimates of the risk of tuberculosis progression in household contacts in the absence of preventive therapy. We used data from a previous study that described the risk of disease after infection among TST-positive household contacts in Australia using an imputation model to remove the effect of the tuberculosis infection treatment.^2^ We estimated risk of progression within 3 years by using the cumulative risk starting 3 months after index patient diagnosis (appendix p 1), assuming that secondary cases developing before that point would be diagnosed during the initial contact evaluation process and could not benefit from preventive therapy. For the 0–17 years age group in the model, we used a weighted average of the progression probabilities and variances for ages 0–4 years (weight 1) and 5–14 years (weight 2). For progression risk in TST-negative household contacts, we used UK data on tuberculosis incidence stratified by TST status (10 mm cutoff)^14^ to parameterise a risk ratio for progression. Because this cohort study used TST results to define the comparison groups, the model parameter derived from this study takes into account the limited sensitivity (estimated to be around 72%) and specificity (estimated to be around 98% in a non-BCG-vaccinated population and 56% in a BCG-vaccinated population) of the TST.^10^

**Table 1:** Model parameters

	**Age group (years)**	**Estimate (IQR)**
**Probability of progression to tuberculosis disease if TST-positive but not sick, 3 months to 3 years after infection**		
Trauer et al, 2016^2^	0–17	0·132 (0·087–0·188)
Trauer et al, 2016^2^	18–34	0·022 (0·016–0·030)
Trauer et al, 2016^2^	35–64	0·022 (0·016–0·030)
Trauer et al, 2016^2^	≥65	0·022 (0·016–0·030)
**Risk ratio for progression to tuberculosis disease in contacts who are TST-negative versus TST-positive**		
Abubakar et al, 2018^14^	All	0·155 (0·132–0·183)
**3 months of weekly isoniazid and rifapentine efficacy among people with positive TST, as OR**		
Zenner et al, 2017^3^	All	0·226 (0·167–0·306)
**4 months of daily rifampicin efficacy among people with positive TST, as OR**		
Zenner et al, 2017^3^	All	0·335 (0·260–0·432)
**6 months of daily isoniazid efficacy among people with positive TST, as OR**		
Zenner et al, 2017^3^	All	0·391 (0·338–0·452)
**Probability of severe adverse event while receiving 3 months of weekly isoniazid and rifapentine**		
Villarino et al, 2015^15^	0–17	0·005 (0·003–0·007)
Unpublished data from authors of Sterling et al^16^	18–34	0·020 (0·018–0·023)
Unpublished data from authors of Sterling et al^16^	35–64	0·042 (0·039–0·045)
Unpublished data from authors of Sterling et al^16^	≥65	0·082 (0·067–0·099)
**Probability of severe adverse event while receiving 4 months of daily rifampicin**		
Diallo et al, 2018^17^	0–17	0·002 (0·001–0·004)
Unpublished data from authors of Menzies et al^18^	18–34	0·008 (0·007–0·010)
Unpublished data from authors of Menzies et al^18^	35–64	0·009 (0·007–0·011)
Unpublished data from authors of Menzies et al^18^	≥65	0·020 (0·012–0·031)
**Probability of severe adverse event while receiving 6 months of daily isoniazid**		
Diallo et al, 2018^17^	0–17	0·002 (0·001–0·003)
Unpublished data from authors of Menzies et al^18^	18–34	0·015 (0·013–0·018)
Unpublished data from authors of Menzies et al^18^	35–64	0·028 (0·025–0·031)
Unpublished data from authors of Menzies et al^18^	≥65	0·053 (0·040–0·068)

TST=tuberculin skin test. OR=odds ratio.

For the effect of different preventive therapy regimens in reducing tuberculosis disease risk, we used the efficacy estimates from a meta-analysis of randomised controlled trials.^3^ We assumed 100% uptake of preventive therapy if prescribed. We did not assume 100% treatment completion, as the efficacy estimates we used reflect the actual treatment completion rates observed in the studies contributing to the meta-analysis. We assumed efficacy to be consistent across age groups. We assumed that the reduction in relative risk of tuberculosis is the same for TST-positive and TST-negative contacts, although the absolute risk reduction is different because the underlying risk of tuberculosis in untreated contacts is different. We conservatively assumed that a person who had a severe adverse event would derive no benefit from preventive therapy.

To obtain age-stratified data on serious adverse events for adults (age ≥18 years), we contacted authors of the 3 months of weekly isoniazid and rifapentine and 4 months of daily rifampicin efficacy trials.^16^, ^18^ The most common adverse event associated with 9 months of daily isoniazid and 4 months of daily rifampicin was drug-induced hepatitis,^18^ whereas the most common adverse event associated with 3 months of weekly isoniazid and rifapentine was hypersensitivity.^16^ For the 3 months of weekly isoniazid and rifapentine trial, we obtained the number and proportion of patients with grade 3–4 adverse events attributed to treatment in the isoniazid-rifapentine group. From the 4 months of daily rifampicin trial, we obtained the number and proportion of patients with grade 3–4 adverse events attributed to treatment and leading to treatment discontinuation from the 4 months of daily rifampicin and 9 months of daily isoniazid study groups. We obtained similar data for children (0–17 years) from published paediatric trials.^15^, ^17^ We assumed that adverse event data for 9 months of daily isoniazid apply to the 6 months of daily isoniazid regimen; we thought this reasonable because in the 3 months of weekly isoniazid and rifapentine efficacy trial, around 90% of the adverse-event-associated discontinuations in the 9 months of daily isoniazid group occurred within 6 months.^19^

Uncertainty in parameters was modelled with appropriate distributions with parameters that were matched to evidence by moment matching (appendix pp 2–4). We report IQRs around mean outcomes, calculated as the 25th and 75th percentiles in model outputs across 10 000 sampled input parameter sets.

### Model outputs

We first modelled the risk of incident tuberculosis disease within 3 years and the risk of a severe adverse event for four age groups (0–17 years, 18–34 years, 35–64 years, and ≥65 years), stratified by TST status, under four treatment conditions (no treatment, 3 months of weekly isoniazid and rifapentine, 4 months of daily rifampicin, and 6 months of daily isoniazid). We then used these outputs to model the risk difference between the two treatment strategies (treat all *vs* treat TST-positive contacts only), by age group, and for populations with different prevalence of TST positivity. Finally, as an illustrative example, we modelled the overall risk difference for hypothetical populations of household contacts with age structure and TST positivity similar to what has been reported from a high-burden setting (New Delhi)^20^ and from a low-burden setting (Amsterdam).^21^ These population parameters are shown in the appendix (p 5). The purpose of this model was not to make treatment recommendations for high-burden and low-burden settings, but rather to illustrate how the same treatment strategy can lead to different consequences when applied to populations with different age structures and TST prevalence.

### Role of the funding source

The funders of the study had no role in study design, data collection, data analysis, data interpretation, or writing of the report. The corresponding author had full access to all the data in the study and had final responsibility for the decision to submit for publication.

## Results

**Figure 2:**
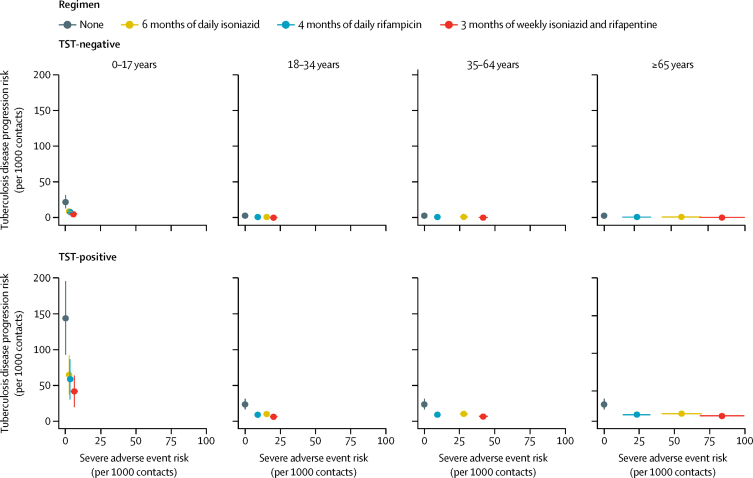
Predicted 3-year risk of tuberculosis disease and risk of severe adverse events among household contacts who do not have tuberculosis disease at initial evaluation, by age group, tuberculin skin test status, and preventive therapy regimen Error bars represent IQRs.

We estimated the risk of incident tuberculosis disease in 3 years and risk of severe adverse events, by age group, TST status, and preventive therapy regimen, among household contacts who did not have tuberculosis disease upon initial evaluation (figure 2). For each 1000 children aged 0–17 years with a positive TST, the number expected to develop tuberculosis in 3 years with no treatment would be 145 (IQR 87–190), compared with 42 (20–54) with 3 months of weekly isoniazid and rifapentine, 59 (29–77) with 4 months of daily rifampicin, and 65 (35–86) with 6 months of daily isoniazid. By contrast, for each 1000 adults aged 18–34 years, the number expected to develop tuberculosis in 3 years with no treatment would be 24 (16–30) compared with seven (3–8) with 3 months of weekly isoniazid and rifapentine, nine (5–11) with 4 months of daily rifampicin, and ten (6–13) with 6 months of daily isoniazid. The tuberculosis risks for other adult age groups were similar to these results.

Table 2 shows the risks of incident tuberculosis disease and severe adverse events expected in different age groups when using different regimens, assuming a 25% prevalence of TST positivity for each age group and comparing a treat-all approach with an approach of treating only TST-positive contacts. For children aged 0–17 years, the number of severe adverse events predicted was lower than the number of tuberculosis cases that would arise without treatment, regardless of treatment approach and regimen. For adults, this remained true only for the treat-TST-only approach, and only for certain age group and regimen combinations. Results assuming different prevalence of TST positivity are shown in the appendix (pp 6–8).

**Table 2:** Risks of incident tuberculosis and severe adverse events incurred by treating all contacts or treating only TST-positive contacts, assuming 25% prevalence of TST positivity, by age and regimen

	**Treat all**		**Treat TST-positive only**	
	Tuberculosis cases per 1000 contacts	Severe adverse events per 1000 contacts	Tuberculosis cases per 1000 contacts	Severe adverse events per 1000 contacts
**0–17 years**				
No preventive therapy	53·3 (37·3–66·5)	0·0 (0·0–0·0)	53·3 (37·3–66·5)	0·0 (0·0–0·0)
3 months of weekly isoniazid and rifapentine	14·9 (8·8–18·8)	5·6 (2·9–7·4)	27·7 (18·2–34·9)	1·4 (0·7–1·9)
4 months of daily rifampicin	21·0 (12·9–26·7)	3·0 (0·9–4·2)	31·9 (21·3–39·7)	0·7 (0·2–1·0)
6 months of daily isoniazid	23·3 (15·2–29·2)	2·5 (0·7–3·5)	33·4 (22·8–41·6)	0·6 (0·2–0·9)
**18–34 years**				
No preventive therapy	8·9 (6·5–10·8)	0·0 (0·0–0·0)	8·9 (6·5–10·8)	0·0 (0·0–0·0)
3 months of weekly isoniazid and rifapentine	2·4 (1·5–3·0)	20·3 (17·6–22·6)	4·5 (3·2–5·5)	5·1 (4·4–5·7)
4 months of daily rifampicin	3·3 (2·2–4·0)	8·8 (7·0–10·3)	5·1 (3·6–6·2)	2·2 (1·7–2·6)
6 months of daily isoniazid	3·7 (2·6–4·5)	15·4 (13·1–17·5)	5·4 (3·9–6·5)	3·9 (3·3–4·4)
**35–64 years**				
No preventive therapy	8·8 (6·6–10·7)	0·0 (0·0–0·0)	8·9 (6·5–10·8)	0·0 (0·0–0·0)
3 months of weekly isoniazid and rifapentine	2·5 (1·6–3·1)	41·8 (38·6–44·9)	4·6 (3·2–5·6)	10·5 (9·7–11·2)
4 months of daily rifampicin	3·3 (2·2–4·1)	9·0 (7·3–10·5)	5·1 (3·6–6·3)	2·3 (1·8–2·6)
6 months of daily isoniazid	3·7 (2·7–4·6)	28·1 (25·2–30·8)	5·4 (4·0–6·5)	7·0 (6·3–7·7)
**≥65 years**				
No preventive therapy	8·8 (6·5–10·7)	0·0 (0·0–0·0)	8·8 (6·5–10·7)	0·0 (0·0–0·0)
3 months of weekly isoniazid and rifapentine	2·8 (1·9–3·4)	84·0 (67·1–98·7)	4·8 (3·4–5·8)	21·0 (16·7–24·7)
4 months of daily rifampicin	3·3 (2·2–4·1)	23·1 (12·0–30·9)	5·1 (3·6–6·3)	5·8 (3·0–7·7)
6 months of daily isoniazid	3·9 (2·7–4·7)	55·1 (40·0–67·6)	5·5 (4·0–6·7)	13·8 (10·0–16·9)

Data are mean (IQR). TST=tuberculin skin test.

**Figure 3:**
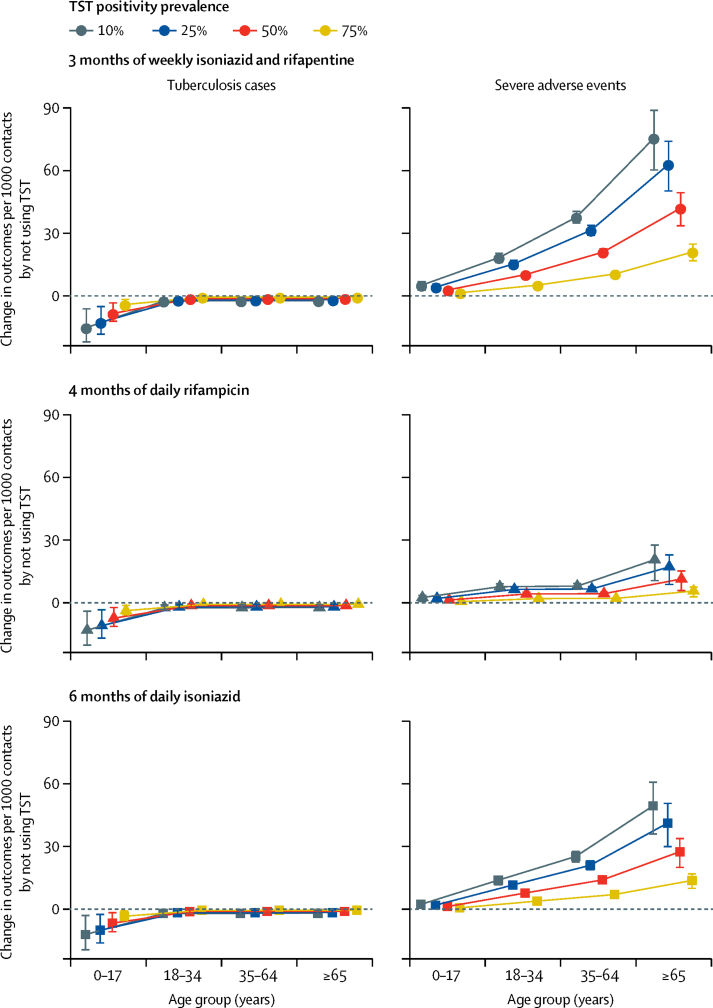
Difference in risks of incident tuberculosis and severe adverse events associated with a treat-all approach versus a treat-TST-only approach, by preventive therapy regimen, age group, and prevalence of TST positivity Error bars represent IQRs. TST=tuberculin skin test.

Figure 3 shows the differences in the risk of incident tuberculosis and the risk of severe adverse events associated with a treat-all strategy compared with a strategy of treating TST-positive contacts. When using 3 months of weekly isoniazid and rifapentine to treat 1000 child contacts aged 0–17 years, 25% of whom had a positive TST, the treat-all approach led to a difference of −13 (IQR −5 to −18) in the number of tuberculosis cases and a difference of four (2 to 6) in the number of severe adverse events compared with treating only those with a positive TST. Results were similar for other regimens (appendix p 9). The treat-all approach led to an increasing number of additional severe adverse events as age increased, with 4 months of daily rifampicin producing the smallest increase. When using 3 months of weekly isoniazid and rifapentine to treat 1000 adult contacts aged 65 years and older, 25% of whom had a positive TST, the treat-all approach led to a difference of −2 (–1 to −3) tuberculosis cases and a difference of 63 (50 to 74) severe adverse events compared with treating only those with a positive TST. By contrast, when using 4 months of daily rifampicin, the treat-all approach led to a difference of −2 (–1 to −3) tuberculosis cases and a difference of 17 (9 to 23) in severe adverse events. As the prevalence of TST positivity increased, the number of TST-negative contacts excluded from the treat-TST-positive strategy decreased; therefore, the differences in outcomes between the two strategies diminished.

Table 3 shows the results of applying these risk differences to hypothetical populations of contacts in high-burden and low-burden settings. In the high-burden setting, a treat-all strategy across all age groups using 3 months of weekly isoniazid and rifapentine led to a difference of −4 (IQR −6 to −2) tuberculosis cases and a difference of nine (8 to 10) severe adverse events per 1000 contacts compared with the strategy of treating only TST-positive contacts. In the low-burden setting, the treat-all strategy with 3 months of weekly isoniazid and rifapentine led to a difference of −3 (–4 to −2) tuberculosis cases and a difference of 38 (34 to 42) in severe adverse events per 1000 contacts.

**Table 3:** Risk differences for incident tuberculosis and severe adverse events for treat-all approach compared with treating only TST-positive contacts in hypothetical household contact cohorts from high-burden and low-burden settings

	**High-burden setting hypothetical cohort**		**Low-burden setting hypothetical cohort**	
	Difference in number of tuberculosis cases per 1000 contacts	Difference in number of severe adverse events per 1000 contacts	Difference in number of tuberculosis cases per 1000 contacts	Difference in number of severe adverse events per 1000 contacts
3 months of weekly isoniazid and rifapentine	−4·2 (−5·7 to −2·1)	9·1 (8·3 to 9·8)	−3·1 (−3·9 to −2·2)	37·9 (33·8 to 41·4)
4 months of daily rifampicin	−3·6 (−5·2 to −1·5)	3·3 (2·7 to 3·8)	−2·7 (−3·6 to −1·8)	10·9 (8·2 to 12·8)
6 months of daily isoniazid	−3·3 (−5·0 to −1·3)	6·2 (5·6 to 6·8)	−2·5 (−3·4 to −1·6)	25·7 (22·0 to 28·8)

Data are mean (IQR). TST=tuberculin skin test.

## Discussion

Treating tuberculosis infection with preventive therapy is a critical component of a comprehensive strategy to eliminate the disease.^22^ When comparing treat-all and treat-TST-positive-only approaches to preventive therapy, we found that outcomes varied on the basis of age, preventive therapy regimen, and prevalence of TST positivity. Among child contacts younger than 18 years, a treat-all approach could avert more cases of tuberculosis disease and would incur a minimal increase in severe adverse events, regardless of preventive therapy regimen and prevalence of TST positivity. For adults, isoniazid-containing regimens were generally associated with more adverse events than rifampicin alone. Moreover, for adults, our model suggests that, since the risk of adverse events increases with age, risk stratification using TST could reduce the risk of treatment-related adverse events, and increased monitoring for adverse events such as hepatoxicity is warranted in older age groups receiving preventive therapy. In weighing up the risks and benefits of widening the delivery of preventive therapy, our results must be considered in light of the fact that, should an adult develop tuberculosis disease, the risk of adverse events during treatment and poor outcomes from treatment also increases with age.^23^ Finally, applying the same risk stratification approach in high-burden and low-burden settings can have different implications in terms of disease and adverse event outcomes, therefore optimal approaches that balance risks and benefits will probably differ across settings.

Our findings could help guide policy decisions around when to make tuberculosis infection tests a prerequisite for preventive therapy among household contacts. In high-burden settings that are already treating contacts younger than 5 years and those with HIV without requiring tuberculosis infection testing, scaling up this recommendation to include older children and adolescents would be a logical first step to expanding the use of preventive therapy, given that the treat-all approach would probably lead to more protection and a minimal increase in adverse events. For adults, our findings give a sense of the anticipated numbers of tuberculosis cases and potential severe adverse events associated with each approach. These results suggest that as treatment is expanded to older age groups, it is crucial for programmes to build systems that can identify and manage adverse events. Moreover, risk stratification using infection testing and the use of non-isoniazid-containing regimens can reduce the number of individuals exposed to toxicity from preventive therapy. Use of IGRA might favour testing-based risk stratification, as current IGRAs have higher sensitivity than TST.^10^ Finally, although our analysis did not evaluate costs, the framework we present could help programmes perform cost assessments to inform policies. Infection testing, administration of preventive therapy, monitoring and managing adverse events, and treating incident tuberculosis cases all incur costs to health-care systems and patients, and these costs vary by context. Knowing the relative numbers of each of these outcomes could help programmes compare local costs of different strategies.

Our modelling exercise complements previous work evaluating preventive therapy strategies. Our model considered only age and TST positivity as risk factors for disease progression; however, the model considered multiple preventive therapy regimens and quantified both tuberculosis disease and adverse event outcomes. Previous studies that used more detailed sets of risk factors to create tools for predicting disease progression have either not considered adverse events or have only considered hepatitis associated with isoniazid preventive therapy.^24^, ^25^ Additionally, research into biomarkers that in the future might provide better predictive value for risk of tuberculosis disease progression than existing tuberculosis infection tests is ongoing.^26^

A major limitation of our approach was that we considered two binary outcomes but did not attempt to quantify their relative importance. In reality, both tuberculosis disease and severe adverse events are associated with a spectrum of patient experiences, and different people might view their relative importance differently.^27^ New methods that jointly analyse competing efficacy and safety risks have recently been proposed for preventive therapy trials,^28^ and could in the future be adapted for modelling a gradient of outcomes.

Another limitation of the outcomes in our model is that they do not capture future consequences of tuberculosis disease. Untreated active disease is linked to tuberculosis transmission in families and communities, impacting people other than the patient. If we had modelled these outcomes, the treat-all approach would probably be more favourable than the treat-TST-positive-only approach given that people who develop tuberculosis disease would be at risk of adverse events from tuberculosis treatment and could contribute to additional cases. We would expect adverse events among the small number of adults treated for active disease to be outnumbered by adverse events among the much larger group receiving preventive therapy, resulting in small changes to our adverse event analysis. Differences in incident tuberculosis would be highly dependent on assumptions made about reproduction number and the timeframe put on the analysis.

Our study was also limited by available data for our model parameters. First, age-stratified data on the natural history of tuberculosis progression following infection were limited, and we used a study from Australia because it had high-quality follow-up data and sought to remove the effect of preventive therapy.^2^ However, disease progression risks might not be the same in high-burden settings where baseline comorbidities and risks of community infection differ. Furthermore, our analysis does not incorporate the effect of sex, HIV status, low body-mass index, diabetes, or large TST reaction size, all of which are associated with increased risk of progression to tuberculosis disease.^29^ Second, we chose to use data on efficacy and adverse events from clinical trials because of high data quality, meaning that our model is limited to drug-susceptible tuberculosis, as results from ongoing trials for preventive therapy for drug-resistant tuberculosis are not yet known. Moreover, outcomes in clinical trials can differ from what is observed in programmatic settings, and increased adherence in clinical trial populations might lead to both greater efficacy and more adverse events than under programmatic conditions. Finally, our model simplistically assumed complete uptake of preventive treatment.

In conclusion, our results suggest that in high-burden settings, making all contacts eligible for preventive therapy will reduce incident tuberculosis in this population. The risk of adverse events can be minimised by using regimens that do not contain isoniazid and, in adults older than 18 years, focusing treatment on individuals with a positive test for infection. Tuberculosis programmes in high-burden settings could use the results of this study to project risks of tuberculosis and severe adverse events, which along with logistical considerations, might help programmes decide when to incorporate tuberculosis infection testing into contact management algorithms. Moreover, future prospective studies nested within programmes that evaluate incident disease, adverse events, and costs could help validate and refine these results, providing a stronger evidence base for programmes to make policy decisions based on their local epidemiological situation.
