# Supplementary material for: Risk-benefit analysis of tuberculosis infection testing for household contact management in high-burden countries: a mathematical modelling study
Source: Lancet Glob Health. 2020 Apr 27;8(5):e672–80. doi: 10.1016/S2214-109X(20)30075-9 (PMC7196883; doi:10.1016/S2214-109X(20)30075-9)
Supplement: Supplementary appendix [file mmc1.pdf]

THE LANCET
Global Health

Supplementary appendix

This appendix formed part of the original submission and has been peer reviewed. We post it as supplied by the authors.

Supplement to: Yuen CM, Seddon JA, Keshavjee S, Dodd PJ. Risk-benefit analysis of tuberculosis infection testing for household contact management in high-burden countries: a mathematical modelling study. *Lancet Glob Health* 2020; **8:** e672–80.

SUPPLEMENTARY MATERIAL

Contents:

- Table S1: TB disease progression risk
- Table S2: Distributional assumptions for model parameters
- Figure S1: Plots of probability distribution functions for model parameters in Table S2
- Table S3: Population age distribution and TST positivity based on published contact cohorts from high- and low-burden settings.
- Table S4: Risks of incident tuberculosis and severe adverse events incurred by treating all contacts or treating only TST-positive contacts assuming 10% prevalence of TST positivity, by age and regimen
- Table S5: Risks of incident tuberculosis and severe adverse events incurred by treating all contacts or treating only TST-positive contacts assuming 50% prevalence of TST positivity, by age and regimen
- Table S6: Risks of incident tuberculosis and severe adverse events incurred by treating all contacts or treating only TST-positive contacts assuming 75% prevalence of TST positivity, by age and regimen
- Table S7: Differences in risks of tuberculosis and severe adverse events associated with a treat-all approach versus a treat-TST-only approach, by preventive therapy regimen, age group, and prevalence of TST positivity

Table S1: TB disease progression risks

Time after infection	Age of contact	Cumulative probability of TB disease	Lower 95% confidence limit	Upper 95% confidence limite
1 month	0-4	0.18	0.13	0.21
1 month	5-14	0.07	0.025	0.12
1 month	15+	0.001	0	0.006
3 month	0-4	0.39	0.26	0.53
3 month	5-14	0.15	0.09	0.22
3 month	15+	0.006	0	0.016
1 year	0-4	0.56	0.41	0.71
1 year	5-14	0.28	0.17	0.35
1 year	15+	0.02	0.01	0.04
3 year	0-4	0.56	0.41	0.71
3 year	5-14	0.28	0.18	0.36
3 year	15+	0.03	0.009	0.05

Data extracted from: Trauer JM, Moyo N, Tay EL, Dale K, Ragonnet R, McBryde ES, et al. Risk of Active Tuberculosis in the Five Years Following Infection . . . 15%? *Chest*. 2016;**149**(2):516-25.

Data extraction was performed using Engauge Digitizer: Mark Mitchell, Baurzhan Muftakhidinov and Tobias Winchen et al, "Engauge Digitizer Software." Webpage: http://markummitchell.github.io/engauge-digitizer, Last Accessed: November 15, 2019

Table S2: Distributional assumptions for model parameters. B = beta distribution; LN = log-normal distribution; E = exponential distribution; parameters named X.33 are the 3-month/3-year variants used in the paper; mqrng = 50% (25%,75%)-iles.

NAME	DISTRIBUTION	DESCRIPTION	SOURCE	mqrng
P.0-17.11	B(13.1447024329769,36.1479316906865)	Progression risk: age 0-17; 1 years - 1 months	Trauer et al	0.263 (0.223 - 0.307)
P.18-34.11	B(5.79496609230769,299.203249292308)	Progression risk: age 18-34; 1 years - 1 months	Trauer et al	0.018 (0.013 - 0.024)
P.35-64.11	B(5.79496609230769,299.203249292308)	Progression risk: age 35-64; 1 years - 1 months	Trauer et al	0.018 (0.013 - 0.024)
P.65+.11	B(5.79496609230769,299.203249292308)	Progression risk: age 65+; 1 years - 1 months	Trauer et al	0.018 (0.013 - 0.024)
P.0-17.31	B(13.1447024329769,36.1479316906865)	Progression risk: age 0-17; 1 years - 3 months	Trauer et al	0.263 (0.223 - 0.307)
P.18-34.31	B(7.27931174746651,243.731438165172)	Progression risk: age 18-34; 1 years - 3 months	Trauer et al	0.028 (0.021 - 0.035)
P.35-64.31	B(7.27931174746651,243.731438165172)	Progression risk: age 35-64; 1 years - 3 months	Trauer et al	0.028 (0.021 - 0.035)
P.65+.31	B(7.27931174746651,243.731438165172)	Progression risk: age 65+; 1 years - 3 months	Trauer et al	0.028 (0.021 - 0.035)
P.0-17.13	B(2.9592822244317,17.6868728297429)	Progression risk: age 0-17; 3 years - 1 months	Trauer et al	0.132 (0.087 - 0.188)
P.18-34.13	B(2.55490051764706,179.9379936)	Progression risk: age 18-34; 3 years - 1 months	Trauer et al	0.012 (0.008 - 0.019)
P.35-64.13	B(2.55490051764706,179.9379936)	Progression risk: age 35-64; 3 years - 1 months	Trauer et al	0.012 (0.008 - 0.019)
P.65+.13	B(2.55490051764706,179.9379936)	Progression risk: age 65+; 3 years - 1 months	Trauer et al	0.012 (0.008 - 0.019)
P.0-17.33	B(2.9592822244317,17.6868728297429)	Progression risk: age 0-17; 3 years - 3 months	Trauer et al	0.132 (0.087 - 0.188)
P.18-34.33	B(4.43579415921528,180.388962474755)	Progression risk: age 18-34; 3 years - 3 months	Trauer et al	0.022 (0.016 - 0.030)
P.35-64.33	B(4.43579415921528,180.388962474755)	Progression risk: age 35-64; 3 years - 3 months	Trauer et al	0.022 (0.016 - 0.030)
P.65+.33	B(4.43579415921528,180.388962474755)	Progression risk: age 65+; 3 years - 3 months	Trauer et al	0.022 (0.016 - 0.030)
RR.TST	LN(-1.86278401275579,0.244747266551745)	Risk ratio for TB given TST-ve vs TST+ve, all ages	Abubakar et al	0.155 (0.132 - 0.183)
OR.3HP	LN(-1.48585291401482,0.446225397965938)	3HP efficacy TST+ as OR, all ages	Zenner et al	0.226 (0.167 - 0.306)
AE.3HP.0-17	B(2.30938119194641,412.609439627759)	4R AE probability, ages 0-17	Villarino et al	0.005 (0.003 - 0.007)
AE.3HP.18-34	B(29.0361139912311,1402.80725720135)	4R AE probability, ages 18-34	Unpublished data from authors of Sterling et al	0.020 (0.018 - 0.023)
AE.3HP.35-64	B(77.2763856730113,1768.87531595417)	4R AE probability, ages 35-64	Unpublished data from authors of Sterling et al	0.042 (0.039 - 0.045)
AE.3HP.65+	B(11.1862673751049,122.188459020377)	4R AE probability, ages 65+	Unpublished data from authors of Sterling et al	0.082 (0.067 - 0.099)
OR.4R	LN(-1.09235617710903,0.376045022775337)	4R efficacy TST+ as OR, all ages	Zenner et al	0.335 (0.260 - 0.432)
AE.4R.0-17	E(332.859141505999)	4R AE probability, ages 0-17	Diallo et al	0.002 (0.001 - 0.004)
AE.4R.18-34	B(12.0807863420021,1371.63389544578)	4R AE probability, ages 18-34	Unpublished data from authors of Menzies et al	0.008 (0.007 - 0.010)
AE.4R.35-64	B(14.0456142322815,1541.27206842236)	4R AE probability, ages 35-64	Unpublished data from authors of Menzies et al	0.009 (0.007 - 0.011)
AE.4R.65+	B(2.36986760235613,100.324395166409)	4R AE probability, ages 65+	Unpublished data from authors of Menzies et al	0.020 (0.012 - 0.031)
OR.6H	LN(-0.93926387468323,0.214350847019905)	6H efficacy TST+ as OR, all ages	Zenner et al	0.391 (0.338 - 0.452)
AE.6H.0-17	E(396.653704743434)	6H AE probability, ages 0-17	Diallo et al	0.002 (0.001 - 0.003)
AE.6H.18-34	B(21.2627981971801,1357.9196121381)	6H AE probability, ages 18-34	Unpublished data from authors of Menzies et al	0.015 (0.013 - 0.018)
AE.6H.35-64	B(45.1939550148373,1568.03374355827)	6H AE probability, ages 35-64	Unpublished data from authors of Menzies et al	0.028 (0.025 - 0.031)
AE.6H.65+	B(6.44144817333538,110.424825828607)	6H AE probability, ages 65+	Unpublished data from authors of Menzies et al	0.053 (0.040 - 0.068)

References for Table S2:

Abubakar I, Drobniewski F, Southern J, Sitch AJ, Jackson C, Lipman M, et al. Prognostic value of interferon-gamma release assays and tuberculin skin test in predicting the development of active tuberculosis (UK PREDICT TB): a prospective cohort study. *The Lancet Infectious Diseases*. 2018;**18**(10):1077-87

Diallo T, Adjobimey M, Ruslami R, Trajman A, Sow O, Obeng Baah J, et al. Safety and Side Effects of Rifampin versus Isoniazid in Children. *The New England Journal of Medicine*. 2018;**379**(5):454-63.

Menzies D, Adjobimey M, Ruslami R, Trajman A, Sow O, Kim H, et al. Four Months of Rifampin or Nine Months of Isoniazid for Latent Tuberculosis in Adults. *The New England Journal of Medicine*. 2018;**379**(5):440-53.

- Unpublished data used in this analysis have since been accepted for publication: Campell JR, Trajman A, Cook VJ, Johnston JC, Adjobimey M, Ruslami R, Eisenbeis L, Fregonese F, Valiquette C, Benedetti A, and Menzies D. Adverse Events Among Adults with Latent Tuberculosis Infection Receiving Daily Rifampicin or Isoniazid: Combined Results of Phase 2 and 3 Randomized Controlled Trials. *The Lancet Infectious Diseases.* pii: S1473-3099(19)30575-4.

Sterling TR, Villarino ME, Borisov AS, Shang N, Gordin F, Bliven-Sizemore E, et al. Three months of rifapentine and isoniazid for latent tuberculosis infection. *The New England Journal of Medicine*. 2011;365(23):2155-66.

Trauer JM, Moyo N, Tay EL, Dale K, Ragonnet R, McBryde ES, et al. Risk of Active Tuberculosis in the Five Years Following Infection . . . 15%? *Chest*. 2016;**149**(2):516-25

Villarino ME, Scott NA, Weis SE, Weiner M, Conde MB, Jones B, et al. Treatment for preventing tuberculosis in children and adolescents: a randomized clinical trial of a 3-month, 12-dose regimen of a combination of rifapentine and isoniazid. *JAMA Pediatrics*. 2015;**169**(3):247-55.

Zenner D, Beer N, Harris RJ, Lipman MC, Stagg HR, van der Werf MJ. Treatment of Latent Tuberculosis Infection: An Updated Network Meta-analysis. *Annals of Internal Medicine*. 2017;**167**(4):248-55.

Figure S1: Plots of probability distribution functions for model parameters in Table S2

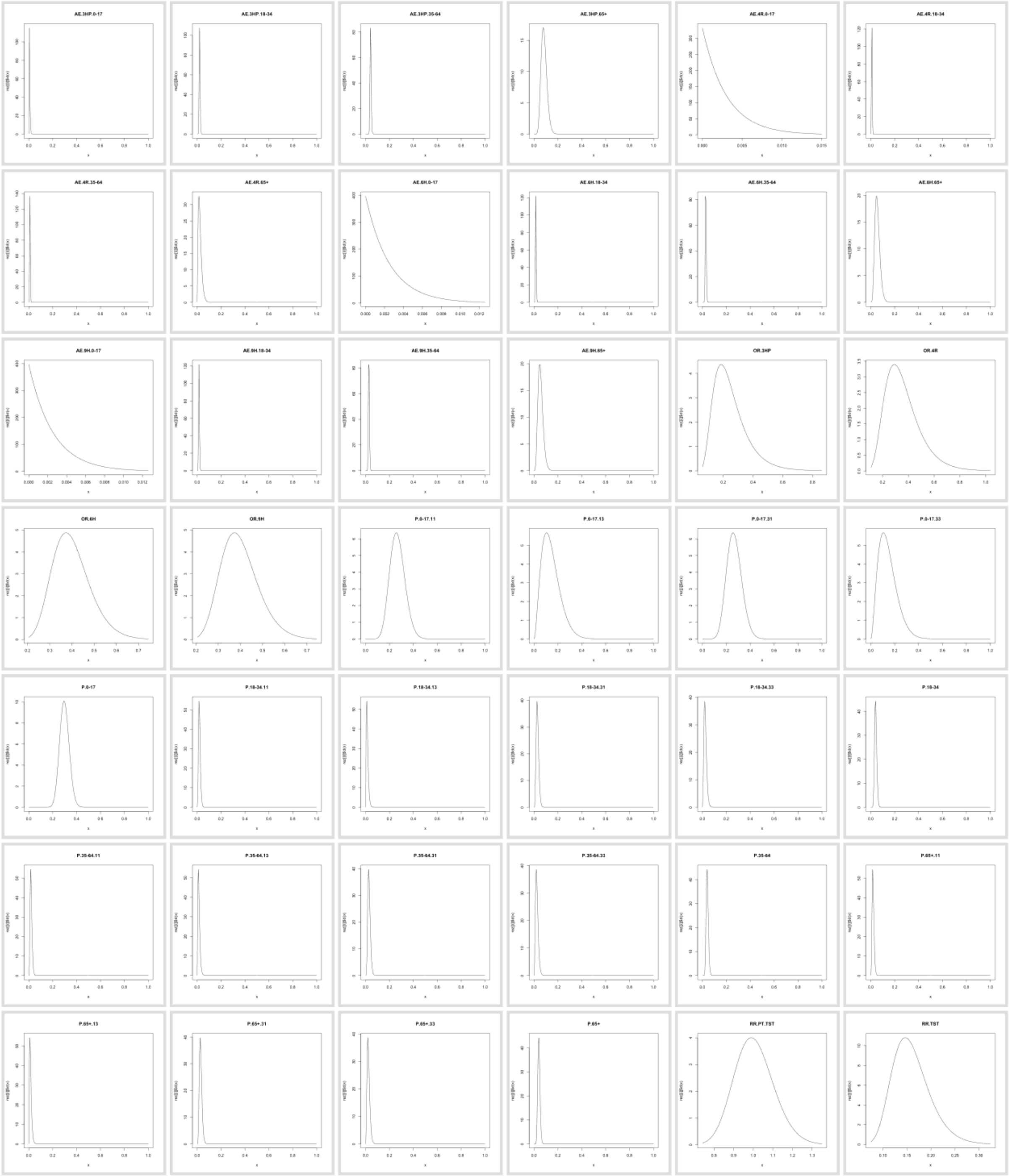

Table S3: Population age distribution and tuberculin skin test positivity based on published contact cohorts from high- and low-burden settings.

Age group (years)	High-burden setting		Low-burden setting	
	Percent of cohort in age group	Percent of age group with positive tuberculin skin test	Percent of cohort in age group	Percent of age group with positive tuberculin skin test
**0-17**	35%	43%	5%	3%
**18-34**	40%	47%	40%	16%
**35-64**	20%	71%	30%	7%
**65+**	5%	70%	25%	9%

These values were based on data published in:

Sharma SK, Vashishtha R, Chauhan LS, Sreenivas V, Seth D. Comparison of TST and IGRA in Diagnosis of Latent Tuberculosis Infection in a High TB-Burden Setting. *PLOS One*. 2017;**12**(1):e0169539.

Sloot R, Schim van der Loeff MF, Kouw PM, Borgdorff MW. Yield of tuberculosis contact investigations in Amsterdam: opportunities for improvement. *European Respiratory Journal*. 2014;**44**(3):714-24.

Table S4: Risks of incident tuberculosis and severe adverse events incurred by treating all contacts or treating only TST-positive contacts assuming 10% prevalence of TST positivity, by age and regimen. TB: tuberculosis disease; TST: tuberculin skin test; AE: adverse event; IQR: interquartile range; PT: preventive therapy; 3HP: 3 months of weekly isoniazid and rifapentine; 4R: 4 months of daily rifampicin; 6H: 6 months of daily isoniazid.

Age group	Regimen	Treat all		Treat TST-positive only	
		TB cases per 1000 contacts (IQR)	Severe AE per 1000 contacts (IQR)	TB cases per 1000 contacts (IQR)	Severe AE per 1000 contacts (IQR)
0-17	No PT	35.1 (24.6, 43.4)	0.0 (0.0, 0.0)	35.1 (24.6, 43.4)	0.0 (0.0, 0.0)
	3HP	9.5 (5.7, 11.9)	5.6 (2.9, 7.4)	24.8 (15.4, 31.4)	0.6 (0.3,0.7)
	4R	13.5 (8.4, 16.9)	3.0 (0.9, 4.2)	26.5 (16.9, 33.4)	0.3 (0.1,0.4)
	6H	14.9 (10.0, 18.6)	2.5 (0.7, 3.5)	27.1 (17.6, 34.0)	0.3 (0.1,0.3)
18-34	No PT	5.9 (4.3, 7.1)	0.0 (0.0, 0.0)	5.9 (4.3, 7.1)	0.0 (0.0, 0.0)
	3HP	1.6 (1.0, 1.9)	20.3 (17.6, 22.6)	4.1 (2.8, 5.1)	2.0 (1.8,2.3)
	4R	2.1 (1.4, 2.6)	8.8 (7.0, 10.3)	4.4 (3.0, 5.4)	0.9 (0.7,1.0)
	6H	2.4 (1.7, 2.9)	15.4 (13.1, 17.5)	4.5 (3.1, 5.5)	1.5 (1.3,1.8)
35-64	No PT	5.8 (4.3, 7.0)	0.0 (0.0, 0.0)	5.8 (4.3, 7.0)	0.0 (0.0, 0.0)
	3HP	1.7 (1.1, 2.0)	41.8 (38.6, 44.9)	4.1 (2.8, 5.1)	4.2 (3.9,4.5)
	4R	2.2 (1.4, 2.7)	9.0 (7.3, 10.5)	4.3 (2.9, 5.3)	0.9 (0.7,1.0)
	6H	2.5 (1.7, 3.0)	28.1 (25.2, 30.8)	4.5 (3.1, 5.4)	2.8 (2.5,3.1)
65+	No PT	5.8 (4.3, 7.1)	0.0 (0.0, 0.0)	5.8 (4.3, 7.1)	0.0 (0.0, 0.0)
	3HP	1.8 (1.2, 2.3)	84.0 (67.0, 98.7)	4.2 (2.8, 5.2)	8.4 (6.7,9.9)
	4R	2.2 (1.5, 2.7)	23.1 (12.0, 30.9)	4.4 (3.0, 5.4)	2.3 (1.2,3.0)
	6H	2.5 (1.8, 3.1)	55.1 (40.0, 67.6)	4.5 (3.1, 5.5)	5.5 (4.0,6.7)

Table S5: Risks of incident tuberculosis and severe adverse events incurred by treating all contacts or treating only TST-positive contacts assuming 50% prevalence of TST positivity, by age and regimen. TB: tuberculosis disease; TST: tuberculin skin test; AE: adverse event; IQR: interquartile range; PT: preventive therapy; 3HP: 3 months of weekly isoniazid and rifapentine; 4R: 4 months of daily rifampicin; 6H: 6 months of daily isoniazid.

Age group	Regimen	Treat all		Treat TST-positive only	
		TB cases per 1000 contacts (IQR)	Severe AE per 1000 contacts (IQR)	TB cases per 1000 contacts (IQR)	Severe AE per 1000 contacts (IQR)
0-17	No PT	83.7 (54.9, 106.8)	0.0 (0.0, 0.0)	83.7 (54.9, 106.8)	0.0 (0.0, 0.0)
	3HP	24.0 (12.9, 30.5)	5.6 (2.9, 7.4)	32.5 (20.4, 40.5)	2.8 (1.4, 3.7)
	4R	33.6 (18.8, 42.9)	3.0 (0.9, 4.2)	40.8 (25.5, 50.9)	1.5 (0.4, 2.1)
	6H	37.2 (22.1, 47.7)	2.5 (0.7, 3.5)	43.9 (28.3, 55.2)	1.3 (0.4, 1.7)
18-34	No PT	13.9 (9.8, 17.1)	0.0 (0.0, 0.0)	13.9 (9.8, 17.1)	0.0 (0.0, 0.0)
	3HP	3.8 (2.2, 4.8)	20.3 (17.6,22.6)	5.2 (3.5, 6.3)	10.2 (8.9,11.4)
	4R	5.1 (3.2, 6.4)	8.8 (7.0,10.3)	6.4 (4.3, 7.8)	4.4 (3.5, 5.2)
	6H	5.8 (3.8, 7.1)	15.4 (13.1,17.5)	6.9 (4.9, 8.4)	7.7 (6.5, 8.8)
35-64	No PT	13.9 (9.7, 17.2)	0.0 (0.0, 0.0)	13.9 (9.7, 7.2)	0.0 (0.0, 0.0)
	3HP	4.0 (2.4, 4.9)	41.8 (38.6,44.9)	5.3 (3.6, 6.5)	20.9 (19.3,22.4)
	4R	5.2 (3.1, 6.4)	9.0 (7.3,10.5)	6.4 (4.2, 7.8)	4.5 (3.7, 5.2)
	6H	5.9 (4.0, 7.3)	28.1 (25.2,30.8)	7.0 (5.0, 8.5)	14.0 (12.6,15.3)
65+	No PT	13.8 (9.7, 17.0)	0.0 (0.0, 0.0)	13.8 (9.7, 17.0)	0.0 (0.0, 0.0)
	3HP	4.4 (2.8, 5.4)	84.0 (67.0,98.7)	5.7 (3.9, 7.0)	41.9 (33.3,49.4)
	4R	5.3 (3.3, 6.5)	23.1 (12.0,30.9)	6.5 (4.4, 7.9)	11.5 (6.1,15.2)
	6H	6.1 (4.1, 7.5)	55.1 (40.0,67.6)	7.2 (5.1, 8.7)	27.5 (19.9,33.6)

Table S6: Risks of incident tuberculosis and severe adverse events incurred by treating all contacts or treating only TST-positive contacts assuming 75% prevalence of TST positivity, by age and regimen. TB: tuberculosis disease; TST: tuberculin skin test; AE: adverse event; IQR: interquartile range; PT: preventive therapy; 3HP: 3 months of weekly isoniazid and rifapentine; 4R: 4 months of daily rifampicin; 6H: 6 months of daily isoniazid.

Age group	Regimen	Treat all		Treat TST-positive only	
		TB cases per 1000 contacts (IQR)	Severe AE per 1000 contacts (IQR)	TB cases per 1000 contacts (IQR)	Severe AE per 1000 contacts (IQR)
0-17	No PT	114.1 (71.8,148.5)	0.0 (0.0, 0.0)	114.1 (71.8,148.5)	0.0 (0.0, 0.0)
	3HP	32.8 (16.1, 42.5)	5.6 (2.9, 7.4)	37.1 (20.3, 47.2)	4.2 (2.1, 5.6)
	4R	45.8 (23.7, 59.4)	3.0 (0.8, 4.1)	49.4 (27.1, 63.1)	2.3 (0.6, 3.1)
	6H	50.8 (29.0, 65.8)	2.5 (0.7, 3.5)	54.1 (32.3, 69.4)	1.9 (0.5, 2.6)
18-34	No PT	18.9 (12.7, 23.7)	0.0 (0.0, 0.0)	18.9 (12.7, 23.7)	0.0 (0.0, 0.0)
	3HP	5.1 (2.9, 6.5)	20.3 (17.7, 22.8)	5.8 (3.6, 7.2)	15.3 (13.3,17.1)
	4R	7.0 (4.0, 8.9)	8.8 (7.0,10.3)	7.6 (4.6, 9.5)	6.6 (5.2, 7.8)
	6H	7.8 (4.9, 9.9)	15.4 (13.0,17.5)	8.4 (5.5, 10.5)	11.6 (9.8, 13.1)
35-64	No PT	19.0 (12.8, 24.0)	0.0 (0.0, 0.0)	19.0 (12.8, 24.0)	0.0 (0.0, 0.0)
	3HP	5.4 (3.1, 6.9)	41.8 (38.6, 44.9)	6.1 (3.8, 7.6)	31.4 (28.9, 33.6)
	4R	7.2 (4.1, 9.0)	9.0 (7.3,10.5)	7.8 (4.7, 9.7)	6.8 (5.5, 7.9)
	6H	8.1 (5.2, 10.1)	28.0 (25.2, 30.7)	8.6 (5.7, 10.7)	21.0 (18.9, 23.0)
65+	No PT	19.1 (13.0, 23.8)	0.0 (0.0, 0.0)	19.1 (13.0, 23.8)	0.0 (0.0, 0.0)
	3HP	6.0 (3.6, 7.5)	83.8 (66.6,98.8)	6.6 (4.2, 8.2)	62.9 (50.0, 74.1)
	4R	7.2 (4.2, 9.1)	22.9 (12.3,30.4)	7.8 (4.8, 9.7)	17.2 (9.2, 22.8)
	6H	8.3 (5.4, 10.5)	55.1 (39.8,67.2)	8.9 (5.9, 11.1)	41.3 (29.8, 50.4)

Table S7: Difference in tuberculosis disease and severe adverse events associated with using a treat-all versus a treat-TST-positive-only approach, by prevalence of TST positivity, age, and regimen. TB: tuberculosis disease; TST: tuberculin skin test; IQR: interquartile range; PT: preventive therapy; 3HP: 3 months of weekly isoniazid and rifapentine; 4R: 4 months of daily rifampicin; 6H: 6 months of daily isoniazid.

Prevalence of TST positivity	Age group	PT regimen	Difference in incident TB cases per 1000 contacts (IQR)	Difference in severe adverse events per 1000 contacts (IQR)
10%	0-17	3HP	-15.3 (-22.1,-6.3)	5.0 (2.6, 6.7)
		4R	-13.0 (-20.1,-3.9)	2.7 (0.8, 3.7)
		6H	-12.2 (-19.5,-3.0)	2.3 (0.7, 3.2)
	18-34	3HP	-2.6 (-3.6,-1.2)	18.3 (15.9, 20.4)
		4R	-2.2 (-3.3,-0.9)	7.9 (6.3, 9.3)
		6H	-2.1 (-3.2,-0.7)	13.9 (11.8, 15.8)
	35-64	3HP	-2.5 (-3.5,-1.1)	37.7 (40.4)
		4R	-2.2 (-3.3,-0.8)	8.1 (6.6, 9.4)
		6H	-2.0 (-3.1,-0.6)	25.3 (22.7, 27.7)
	65+	3HP	-2.4 (-3.5,-1.0)	75.6 (60.3, 88.8)
		4R	-2.1 (-3.3,-0.8)	20.8 (10.8, 27.8)
		6H	-2.0 (-3.1,-0.6)	49.6 (36.0, 60.9)
25%	0-17	3HP	-12.8 (-18.4,-5.3)	4.2 (2.2, 5.6)
		4R	-10.8 (-16.8,-3.2)	2.2 (0.6, 3.1)
		6H	-10.1 (-16.2,-2.5)	1.9 (0.5, 2.6)
	18-34	3HP	-2.1 (-3.0,-1.0)	15.2 (13.2, 17.0)
		4R	-1.9 (-2.8,-0.7)	6.6 (5.2, 7.7)
		6H	-1.7 (-2.6,-0.6)	11.6 (9.8, 13.1)
	35-64	3HP	-2.0 (-2.9,-0.9)	31.4 (29.0, 33.7)
		4R	-1.8 (-2.7,-0.7)	6.8 (5.5, 7.9)
		6H	-1.7 (-2.6,-0.5)	21.0 (18.9, 23.1)
	65+	3HP	-2.0 (-2.9,-0.8)	63.0 (50.2, 74.0)
		4R	-1.8 (-2.7,-0.6)	17.4 (9.0, 23.2)
		6H	-1.6 (-2.6,-0.5)	41.3 (30.0, 50.7)
50%	0-17	3HP	-8.5 (-12.3,-3.5)	2.8 (1.4, 3.7)
		4R	-7.2 (-11.2,-2.2)	1.5 (0.4, 2.1)
		6H	-6.8 (-10.8,-1.7)	1.3 (0.4, 1.8)
	18-34	3HP	-1.4 (-2.0,-0.7)	10.1 (8.8)
		4R	-1.2 (-1.8,-0.5)	4.4 (3.5, 5.2)
		6H	-1.2 (-1.8,-0.4)	7.7 (6.5, 8.8)
	35-64	3HP	-1.4 (-1.9,-0.6)	20.9 (19.3, 22.4)
		4R	-1.2 (-1.8,-0.5)	4.5 (3.7, 5.2)
		6H	-1.1 (-1.7,-0.3)	14.0 (12.6, 15.4)
	65+	3HP	-1.3 (-1.9,-0.6)	42.0 (33.5, 49.4)
		4R	-1.2 (-1.8,-0.4)	11.6 (6.0, 15.4)
		6H	-1.1 (-1.7,-0.3)	27.5 (20.0, 33.8)
75%	0-17	3HP	-4.3 (-6.1,-1.8)	1.4 (0.7, 1.9)
		4R	-3.6 (-5.6,-1.1)	0.7 (0.2, 1.0)
		6H	-3.4 (-5.4,-0.8)	0.6 (0.2, 0.9)
	18-34	3HP	-0.7 (-1.0,-0.3)	5.1 (4.4, 5.7)
		4R	-0.6 (-0.9,-0.2)	2.2 (1.7, 2.6)
		6H	-0.6 (-0.9,-0.2)	3.9 (3.3, 4.4)
	35-64	3HP	-0.7 (-1.0,-0.3)	10.5 (9.7, 11.2)
		4R	-0.6 (-0.9,-0.2)	2.3 (1.8, 2.6)
		6H	-0.6 (-0.9,-0.2)	7.0 (6.3, 7.7)
	65+	3HP	-0.7 (-1.0,-0.3)	21.0 (16.7, 24.7)
		4R	-0.6 (-0.9,-0.2)	5.8 (3.3, 7.7)
		6H	-0.5 (-0.9,-0.2)	13.8 (10.0, 16.9)
